# Role of Placental Extracts in Periodontal Regeneration: A Literature Review

**DOI:** 10.7759/cureus.26042

**Published:** 2022-06-17

**Authors:** Laxmi Jaahnavi Devarampati, Rekha R Koduganti, Sharmika Savant, Pranavi Gullapelli, Swetha Manchala, Akhila Mydukuru

**Affiliations:** 1 Department of Periodontics, Panineeya Mahavidyalaya Institute of Dental Sciences and Research Centre, Hyderabad, IND

**Keywords:** tissue engineering, placentrex gel, chorion membrane, amnion membrane, periodontal regeneration

## Abstract

Periodontium is a specialized tissue surrounding the teeth. It is made up of the gingiva, periodontal ligament, cementum, and alveolar bone. The healing of periodontal tissues when infected occurs through repair and regeneration. The central dogma of regenerative periodontics is to stimulate a cascade of healing events that, if coordinated well, can lead to proper tissue synthesis which in turn would play a very important part in managing periodontitis and preventing tooth loss.

Many regenerative procedures are being followed in periodontics using newer and modified barrier membranes. Placental membranes like amnion, chorion and amnion-chorion membranes are one among these that serve the purpose because of their active components and therapeutic effects. This literature review highlights the benefits of placental extracts in regenerative periodontal therapy.

## Introduction and background

The placenta is an organ developed during pregnancy which is enriched with mesenchymal stem cells (MSCs) and its presence is vital for fetal growth and maturation. These MSCs have the capacity to differentiate into different cell types and also have the added advantage of renewing themselves, which places them in high demand for regenerative procedures. The fresh membrane is extracted from the placenta during delivery via the vaginal or caesarean procedure. The cleaned membrane is placed in a 0.025 per cent solution of sodium hypochlorite and kept at 4°C in a sterile solution containing penicillin. Placental membranes can remain sterile for up to six weeks [1].

Cultured whole human amniotic membrane is a source of pluripotent stem cells which help in the formation of the primitive liver, lung, neural, epithelial, haematopoietic cells and digestive tract. The human amnion‑derived cells are capable of forming cells of all three germ layers [2]. The advances in the cell therapy approach combined with the option of auto banking provide us with a scope of using placental extracts in regenerative therapies [3]. The use of foetal membranes for skin transplantation has been in vogue since 1910 [4]. These membranes have been utilised in ophthalmology as well [5]. In animals afflicted with diabetes, it has been noticed that when these placental extracts were administered it led to accelerated healing of wounds which prompted their use in diabetic neuropathy and angiopathy [6]. The treatment of nonhealing trophic ulcers, enterocutaneous fistula, orthopaedic pathology and the like has been performed using amnion membrane transplantation [7]. The placental extract also contains nucleotides like polydeoxyribonucleotides (PDRNs), that are known for their regeneration. Thus, amnion or amniotic membrane (AM), chorion membrane, amnion chorion membrane (ACM) and gel forms of placental extracts have multiple applications in medicine and dentistry. Cryopreserved, dehydrated amnion-chorion laminate is the available source of placental allograft for dental use [8].

The loss of soft and hard tissues is the ultimate outcome of periodontitis and thus restoring these tissues back to health is of prime importance. The concept of guided tissue regeneration (GTR) and guided bone regeneration (GBR) have thus been used as treatment approaches and placental extracts have also carved a niche for themselves in regenerative medicine owing to their biological properties.

## Review

Properties of placental extract membranes

Amnion and chorion membranes constitute the fetal component of extraembryonic tissue. The outer layer of the AM is the chorion membrane (CM). The AM consists of three layers: the epithelium, basement membrane, and the stroma which consist of an inner compact layer, middle fibroblast layer, and outermost spongy layer [9]. The amniotic epithelial cells are in contact with the basement membrane whereas the amniotic MSCs are located in the deeper spongy layer of the membrane [10]. Chorion is composed of a reticular layer, the basement membrane, and trophoblasts. The AM is 0.02-0.5mm thick and its self-adherent property permits it to intimately adapt over the root contours and defect areas [8]. The resorption rate of BioXclude ACM (Snoasis Medical, Golden, CO) was 8-12 weeks, whereas a dehydrated CM resorbed in two to four weeks [11].

As the reticular layer interacts with the spongy layer, the chorion is three to four times thicker than the amnion. Collagens I, III, IV, V, and VI are present in the reticular network [12]. The amniotic membrane has an inhibitory effect on macrophages and polymorphonuclear neutrophils [13], Interleukin 1𝛼and interleukin 1β [14], due to which its anti-inflammatory action is potentiated.

The ligands for CD44 are the glycosaminoglycans and hyaluronic acid present in the amniotic membrane which facilitate the adhesion and entrapment of inflammatory cells including lymphocytes onto their surface [15].

The wounds treated with AM heal by regeneration as the membrane downregulates transforming growth factor 𝛽 and its receptor expression by fibroblasts. Human amniotic membrane (HAM) has been tried in the reconstruction of temporomandibular joint ankylosis due to its antifibrotic property [16,17].

The AM facilitates the migration of epithelial cells, reinforces basal cell adhesion, promotes epithelial differentiation, prevents epithelial apoptosis, and promotes epithelialization in the healing of wounds. Their good permeability in contrast to other synthetic materials provides sufficient oxygenation for epithelial cells [18-22].

Transplantation of fresh AM resulted in the expression of HLA I antigens causing a mild inflammatory response [23] which was not observed with the cryopreserved amniotic membrane where the epithelial cells are lost during the process of cryopreservation [24]. Thus, cryopreserved tissue grafts of placental membrane materials have a low risk of immune rejection [25].

The AM produces 𝛽-defensins, especially 𝛽3-defensin, which inhibits the release of MMPs and suppresses proteinase action, thus leading to decreased inflammatory cell infiltration [26,27]. The apoptosis of leukocytes is promoted by the amniotic membrane and due to the presence of interstitial collagens in it, the membrane is resistant to proteolytic factors [28,29]. The low molecular weight elastase and proteinase inhibitors and elafins, which are part of the innate immune system are responsible for the antimicrobial property of AM [30].

The ACM provides a biomatrix with various proteins like lactoferrin, laminin-5, platelet-derived growth factor (PDGF) α, and β, fibroblast growth factor; transforming growth factor-β. collagen types I, III, IV, V, and VI aid in wound healing. Its antiviral property is due to the presence of cystatin E, an analogue of cysteine proteinase inhibitor [31-34].

The amniotic membrane works as an excellent scaffold, providing the ideal environment for the growth and differentiation of cells. Due to the rapid angiogenesis which occurs the grafted area heals rapidly and uneventfully. Peptides present in the placental extract including fibronectin III, regulate trypsin activity. It has been demonstrated that one or more peptides from human placental extract including fibronectin type III, help in the regulation of trypsin activity which aids in debridement and prevents keloid formation during wound healing [35].

Clinical applications 

BioXclude is a second-generation placental allograft composed of amnion and chorion tissue (300um in thickness) and has been used in GTR and GBR procedures. It has the property of self-adherence thus suturing is avoided, which in turn reduces the operating time. Hence this material is the preferred option in recession defects, particularly in the posterior region.

Ambio5™ (Katena, Denville, NJ) is a third-generation amniotic membrane, which is thicker and more amenable for transplantation and has yielded good results.

Another study was conducted on 15 patients with 30 mandibular degree II furcation defects, who were randomly allotted into Group I (PRF and AM) and Group II (PRF only). The clinical parameters like PI, GI, PPD, RAL, and furcation depth were assessed at baseline, three months, and six months. The PRF+ amnion group showed better improvement in the treatment of grade II furcation [36].

A study was conducted on 16 patients with class I gingival recession. Patients were divided into two groups with those allocated in group I undergoing CAF +AM and those allotted in group II undergoing CAF with PRF. The clinical parameters were evaluated at six and 18 months postoperatively. It was concluded that CAF with AM and CAF with PRF were equally effective in providing clinically significant outcomes, however, with respect to root coverage the AM showed a better percentage of root coverage as compared to PRF [37].

Fifty-one subjects with bilateral class I gingival recession defects were randomly divided into two groups, wherein the test group was treated with an amniotic membrane and coronally positioned flap, while the control group was treated with a coronally positioned flap alone. Clinical parameters such as RD, RW, PD, RAL, WKG, and TKG were recorded at baseline and after five years of follow-up. Intergroup comparison showed a non-significant difference in all variables except the TKG. This study concluded that AM helped improve the TKG, which is beneficial in the maintenance of the gingival margin [38].

In a patient aged 40 years, a combination of bilaminar and CAF techniques with HAM was performed to treat a class I gingival recession. The study results favoured the use of HAM as the recession was completely covered, moreover, there was an improvement in the gingival phenotype [39].

Fifty patients with PPD ≥ 6 mm and an intra-bony component of ≥ 3 mm were randomly allocated to collagen membrane and biphasic calcium phosphate group as well as amniotic membrane and biphasic calcium phosphate groups. It was inferred that both the groups performed equally well and that an amniotic membrane with biphasic calcium phosphate could be preferred as a treatment option for intra-bony defects [40].

The anti-inflammatory effect of chorion as a barrier membrane in periodontal pocket therapy was evaluated by assessing interleukin-11 (IL-11) level in gingival crevicular fluid (GCF). Two sites in two quadrants from each of the 10 patients were selected and randomly allocated in Group 1 (flap surgery) and Group 2 (flap surgery and chorion membrane placement). Intergroup comparison showed a statistically significant decrease in SBI, PPD, CAL, and IL-11 in Group 2 compared to Group 1 at four weeks. It was concluded that adjunctive use of chorion membrane in flap surgery was effective in treatment outcomes [41].

Eighteen intra-bony defects in 9 patients with chronic periodontitis were randomly assigned to group 1 (FDBA and chorion membrane) and group 2 (DFDBA and chorion membrane) for periodontal therapy. Clinical and radiographic (RVG) measurements were made at baseline and 12 months. The results were not statistically significant, however, group 1 (FDBA) showed an increase in bone density which was statistically significant. Within the limitations of the present study, both the groups showed similar results with a significant increase in bone density in the FDBA group [42].

In a study 30 sites with Miller's Class, I and class II recession were taken and randomly allocated to the chorion membrane (test) PRF membrane (control) group. The clinical parameters recorded were CAL, REC-HT, REC-WD, WKG, and GTH. It was observed that there was a significant improvement in all the parameters in both the groups, however, on intergroup comparison the test group (chorion membrane) showed better results related to CAL, REC-HT, and GTH when compared to the control group [43].

In a case report a patient had a faulty post and core with a crown with respect to the maxillary right central incisor with a PD of 8 mm and HGR 2.5 mm. After the crown was replaced, a flap was reflected by a semilunar incision and after debridement, a chorion membrane was placed and sutured back. The patient was evaluated at different points of time postoperatively and it was observed that all the clinical parameters improved over a period of six months [44].

Around 30 patients with Miller's Class I and class II GR-type defects were divided into three groups randomly. In Group A 10 patients were treated with only CAF, group B 10 patients were treated with CAF, CM and DFDBA. Similarly, in Group C 10 patients were treated with CAF, AM and DFDBA. Clinical parameters were assessed at baseline and three months postoperatively. The percentage of root coverage obtained within the study groups was highest in group B [11].

A cohort of 20 patients with 25 Class I/II interdental papillary recession defects were treated with ACM and coronal advancement of the gingiva papillary unit via a semilunar incision on the labial aspect followed by a sulcular incision within the area of interest. The black triangle height (BTH) and also the black triangle width (BTW) were calculated by image analysis software, which showed a statistically and highly significant difference from the baseline until three and six months postoperatively. It was inferred that ACM allograft in conjunction with a coronally advanced flap can be an appropriate minimally invasive alternative for papillary regeneration [45].

A study was done to compare treatments of the deficient ridge with ACM/dPTFE membranes in 22 non-molar sites on the identical arch. Postoperative clinical and radiographic ridge dimensions weren't significantly different between the two treatments. ACM sites had significantly more osteoid and better bone volume density compared with dPTFE. Additionally, the researchers remarked that ACM use may improve both patient and clinician-concerned outcomes related to implant placement [46].

Dehydrated amnion/ chorion membrane allograft allows it to be administered as a topical powder or mixed with saline to create an injectable solution or a topical gel. Local injection of Placentrex is a very good therapeutic option when administered in the early stages of oral submucous fibrosis without any side effects and contraindications [47].

Compared to povidone-iodine and saline dressing Placentrex gel provided faster healing without causing interference to granulation tissue and was found to be effective even in presence of pus serum, blood, and slough [48].

Another study was done to evaluate the healing efficacy of topically applied placental extract gel both clinically and histologically.10 healthy patients in the age group of 18-35 years. who were indicated for depigmentation procedure were selected for the study. Depigmentation was done with the scalpel technique on the maxillary and mandibular anterior region. In group, A human placental extract gel was applied to the wound and a non-eugenol pack was placed whereas group B was covered with a non-eugenol pack only. Wound Healing Index and Visual Analogue Score were assessed after seven and 15 days. The epithelisation of the wound was assessed by using toluidine blue after seven days of surgery. Application of human placental extract (HPE) gel showed a statistically significant improvement clinically and histologically in Group A with a distinct parakeratinized stratified squamous epithelium with fibrous connective tissue and nil inflammatory cell infiltrates. Whereas in Group B eight individuals showed moderate inflammatory cell infiltrate. It was concluded that local administration of HPE directly onto wound margins promotes wound healing due to an increase in the amount of transforming growth factor in the early phase of wound healing and vascular endothelial growth factor in the late phase [49]. After depigmentation, it has been observed that the application of Placentrex gel helped in healing the wound better with better patient comfort when compared to a periodontal dressing alone [50].

Amniotic membrane in tissue engineering

The scaffold is one of the triads required for tissue engineering. It has to be biocompatible with the host tissue to help in regeneration. The amniotic membrane is enriched with cytokines and growth factors which augment its benefits when used as a three-dimensional scaffold for sustained drug release or to initiate attachment of cells required for regeneration.

## Conclusions

In this review, the benefits of using placental extracts were highlighted. As periodontal regeneration revolves around the presence of cells, signaling molecules, and scaffolds, the advent of amnion and chorion membranes has largely benefitted the field of regenerative periodontics, as both these membranes have a vast array of growth factors to promote healing and they can also work as biocompatible scaffolds to orchester the ingrowth of cells and blood vessels to initiate regeneration and repair. Many more studies should be done using these membranes to validate their role in regenerative medicine.
